# Rehabilitation in Patients Diagnosed with Arthrogryposis Multiplex Congenita: A Systematic Review

**DOI:** 10.3390/children10050768

**Published:** 2023-04-24

**Authors:** Catalina E. García Aguilar, Cristina García-Muñoz, Ines Carmona-Barrientos, Maria Jesus Vinolo-Gil, Francisco Javier Martin-Vega, Gloria Gonzalez-Medina

**Affiliations:** 1 Hospital Virgen de las Montañas, C. P.º Ambulatorio, Villamartin, 11650 Cadiz, Spain; 2 Department of Nursing and Physiotherapy, University of Cadiz, 11009 Cadiz, Spain; 3 Rehabilitation Clinical Management Unit, Interlevels-Intercenters Hospital Puerta del Mar, Hospital Puerto Real, Cadiz Bay-La Janda Health District, 11006 Cadiz, Spain; 4 Department Biomedical Research and Innovation Institute of Cadiz (INiBICA), Research Unit, Puerta del Mar University Hospital, University of Cadiz, 11009 Cadiz, Spain; 5 CTS-986 Physical Therapy and Health (FISA), University Institute of Research in Social Sustainable Development (INDESS), 11009 Cadiz, Spain

**Keywords:** arthrogryposis multiplex congenita, rehabilitation, physical therapy

## Abstract

Arthrogryposis multiplex congenita is a condition characterised by contractures and deformity in two or more body areas. Physiotherapy may be an appropriate treatment. The aim was to systematically review the evidence for rehabilitation in arthrogryposis multiplex congenita. A systematic review was performed following the PRISMA 2020 criteria. The search was conducted in PubMed, ScienceDirect, Scielo, Scopus, Web of Science, ENFISPO, JSTOR, Google Scholar, ProQuest, Cochrane Library and PEDro from inception until October 2022. To assess the methodological quality, we used the different aspects of the critical appraisal tool JBI. We included 14 studies (6 case reports, 5 case series, 2 cross-sectional and 1 qualitative study). Sample sizes ranged from 1 to 50 participants, with an age range between 11 days and 35 years. Most studies employed multicomponent therapy, mainly kinesitherapy, massage therapy, use of physical agents and stretching, some combined it with orthopaedic therapy, or it was complementary to surgical treatment. The key to improving the clinical picture was early and individualised care, tailored to the characteristics of the patients. Regarding methodological quality, the main conflicts encountered were in the reporting of participant characteristics and experimental interventions. Rehabilitation provides satisfactory results in the treatment of arthrogryposis multiplex congenita. More scientific production and randomised clinical trials are needed.

## 1. Introduction 

Arthrogryposis multiplex congenita (AMC) appears during the embryonic period and can be defined as a non-progressive syndrome presenting with multiple congenital contractures in at least two body areas [1]. Although the aetiology remains unknown, genetic environmental factors and problems during foetal development seem to be directly involved. Globally, it affects 1 in 5000 new-borns each year. In addition to joint contractures, the clinical picture is variable, with frequent physical and cognitive disorders affecting the daily life of AMC patients. The clinical picture of AMC is diverse; at the physical level, these people may present contractures, limitations in joint range, alteration of muscle strength and spinal deformities. On a systemic level, we could find alterations in the central nervous, respiratory, gastrointestinal and genitourinary systems [2]. Taking into account all these manifestations, physiotherapy as a rehabilitative strategy could be considered vital, as it could provide a comprehensive approach to people with AMC.

The approach to patients with AMC requires a multidisciplinary team in which rehabilitation, mainly through physiotherapy, will be fundamental. Thanks to rehabilitation, we can prevent the progression of the symptoms of arthrogryposis, as well as improve the autonomy and functionality of patients so that they can be as independent as possible.

Rehabilitation treatment can be very broad, so it is important to determine which techniques are most effective and with which the best results are obtained, for which it is necessary to carry out a systematic review of the literature. The scientific literature has reviewed the effectiveness of physiotherapy in temporomandibular disorders in people with AMC, showing improvements in mobility, swallowing, speech and breathing [3]. The scoping review by Ganong et al. [4] reviews the use of surgical techniques and some rehabilitation programmes for muscle and joint function in patients with AMC.

However, there is a need to review the therapeutic options and their effects on other variables beyond muscle or joint function. This has not been performed so far or, at least, the authors of this study have not found evidence of it. We therefore propose to undertake a review to facilitate the understanding and rehabilitative treatment of this disease to mark a starting point for researchers and health professionals involved in the study and treatment of this disease who, until now, have had no feedback on the existing evidence on this subject.

Therefore, the main objective of this study is to systematically find and analyse, for the first time, the published scientific literature on the evidence related to the benefits of the use of rehabilitative treatments used to date in arthrogryposis. We hypothesise that these rehabilitative treatments will provide benefits compared to no treatment. 

## 2. Materials and Methods

### 2.1. Design

A systematic review was conducted following the Preferred Reporting Items for Systematic Reviews and Meta-analyses (PRISMA 2020) criteria (18). This review was registered in the OSF registry with the identifying link (https://doi.org/10.17605/OSF.IO/VSZTQ, (accessed on 3 October 2022)). 

### 2.2. Search Strategy

Two independent reviewers (C.E.G.A. and G.G-M) carried out the search in the databases: PubMed, ScienceDirect, Scientific Electronic Library Online (Scielo), Scopus, Web of Science (WoS), Library of the Faculty of Nursing, Physiotherapy and Podiatry of the Complutense University of Madrid (ENFISPO), Journal Storage (JSTOR), Google Schoolar, Library of University of Cadiz, ProQuest Research Library, Cochrane Library, Elton Bryson Stephens Company and Physiotherapy Evidence Database (PEDro), from their inception until October 2022.

The following MeSH terms: Arthrogryposis, Physical Therapy Modalities, Contracture, Rehabilitation, Joints, Clubfoot; and descriptors in health sciences DeCS: Arthrogryposis, Physical Therapy, Arthrogryposis multiplex congenita, treatment, Physical Therapy, Fisioterapia, Artrogriposis, Artrogriposis múltiple congénita, tratamiento and contractura were employed. Terms such as contraturas, joint or clubfoot were used because they are the most prevalent features of this pathology [1]. These terms were combined using the Boolean operators “AND” and “OR”. All information about the complete search strategy and results can be seen in Table 1.

A grey literature search was conducted. However, the studies found were not of sufficient quality to be included in our study.

### 2.3. Inclusion and Exclusion of Studies

The search was based on the research question PICO (Patients, Intervention, Comparison and Outcomes) [5]. 

The inclusion criteria were:

1. People diagnosed with arthrogryposis multiplex congenita (AMC) of every age;
2. Rehabilitation interventions or studies that reports results related to rehabilitation approaches;
3. Any type of comparator (e.g., early treatment versus late treatment, multidisciplinary approach versus single approach, physiotherapy versus other treatments);
4. Health status, joint contractures, joint deformities or independence in activities of daily living;
5. Any type of study, in any language.

The exclusion criteria were:

1. Any associated neuromuscular disease;
2. Any design of study that do not report results from included participants (e.g., protocol of study);
3. Interventions focused only on surgery.

### 2.4. Study Selection Process

Two independent reviewers (G.G-M and I.C.-B.) conducted the initial search in which the total number of records identified in the search was calculated. Published records were located in each of the databases. The total number of records screened was noted, in addition to the deleted records, after reading the title and abstract. 

Once the duplicates had been eliminated, a selection by title and abstract was made before the manuscripts were read in full. Those studies that met the eligibility criteria were included in this review. If there was any debate, a consensus was reached with the corresponding author (C.G-M).

For the descriptive analysis of the data, a table was prepared with the following data: authors and year, type of study, participants, intervention, measurement tools, variable and results.

### 2.5. Assessment of Methodological Quality

Two independent reviewers (C.E.G.A. and C.G-M) assessed the quality and methodological validity of the selected studies. Due to the wide variety of study types, the different versions of the Critical Appraisal JBI tool were used [6]. 

JBI tools allow the methodological quality of the following types of studies to be assessed: cross-sectional studies, case control studies, case reports, case series, cohort studies, diagnostic test accuracy, economic evaluation, prevalence studies, qualitative research, quasi-experimental studies, randomised controlled trials and systematic reviews, both text and opinion. Since the aim of this review was to ascertain the existing literature on the therapeutic approach based on rehabilitation in patients with AMC, we did not set our own inclusion criteria for a single type of study. Therefore, the JBI tool was selected to obtain homogeneity in the assessment of methodological quality due to its wide catalogue for the different types of studies. 

The objective of the JBI tools is to assess the methodological quality of a study and to determine the extent to which a study has addressed the possibility of bias in its design, conduct and analysis. All JBI tools are assessed through a series of items related to the methodology of the different studies. Each item is answered with “yes”, “no”, “unclear” and “not applicable”. At the end of each tool, there is the possibility to include an overall assessment of the methodological quality of the study.

### 2.6. Data Synthesis

Results were reported descriptively. Meta-analysis was not applicable due to heterogeneity of studies, variables and the limited data from the primary studies.

## 3. Results

### 3.1. Study Selection

**Figure 1:**
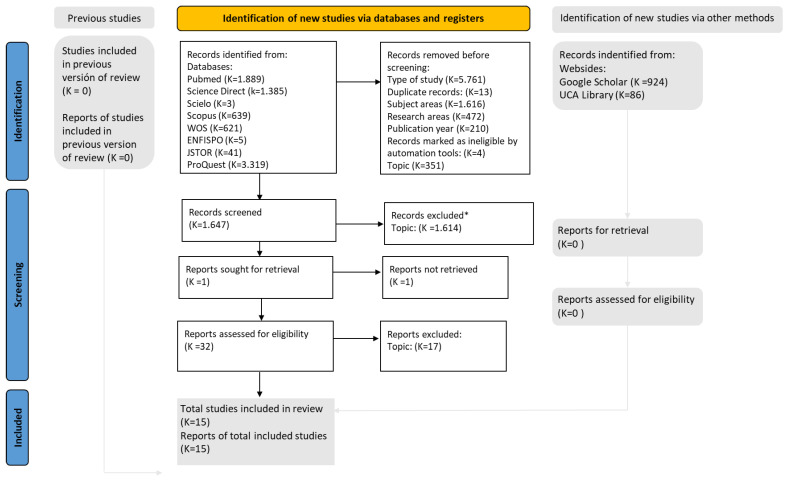
Flow diagram PRISMA 2020 of study selection. * Excluded by title and abstract.

Figure 1 shows the flow diagram of study identification and selection and the reasons for excluding studies. The search strategy identified 7.743 records, of which 15 studies with a total of 212 patients were included for review. Six case reports [7,8,9,10,11,12], five case series studies [13,14,15,16,17], two cross-sectional studies [18,19], one qualitative methodology study [20] and one pilot study were included. 

### 3.2. Study Characteristics

Most of the patients included in each of the studies were young, mostly infants [7,8,10,11,16,18], but nine adult patients were also included [17,20]. All patients studied were diagnosed with arthrogryposis multiplex congenita. Most were infants or young children. They had no cognitive or communication problems and their Intelligence Quotient was normal. However, due to the idiosyncrasies of the disease, all participants had multiple contractures and deformities.

The sample size of all studies analysed ranged from approximately 1 to 50 participants. The age ranged from an 11-day-old new-born to a 35-year-old woman. In terms of sex, both boys and girls were assessed. The total study time ranged from 7 days to 156 months. 

The main objectives of the study were to achieve maximum range of movement in all the patient’s joints and improve their quality of life, functionality and independence. 

The variables are also very diverse and different tools are used to measure them: scales, questionnaires, graphs or diagnostic tests. Even so, we note that, within the deformity variable, the most studied is the congenital clubfoot associated with arthrogryposis, or in other words, clubfoot. Numerous studies have been carried out on the typical position of these feet and Ponseti’s method has been shown to be very effective for early correction and to reduce the need for surgical treatment.

Most of the studies detailing the rehabilitation intervention describe a multicomponent type of intervention, where the basis is the adaptation of the intervention to meet the needs of the individual patient. The interventions consisted of strengthening and stretching exercise programmes to be performed at home, use of splints, functional orthoses and casts, passive kinesitherapy, electrotherapy, kinesiotape, stimulation of motor development patterns through mat exercises, massage to relax arthrogryposis contractures, respiratory physiotherapy protocols and gait re-education. Some of them combine physiotherapy, occupational therapy, hydrotherapy, psychotherapy or even art therapy. The main characteristics of the above-mentioned interventions are listed in Table 2.

Within physiotherapy programmes, one of the most important techniques is kinesitherapy, as it increases the range of motion and flexibility of structures [11,21]. Gentle and progressive passive mobilisations of all joints are used, as well as painless active mobilisations, which work on the weakest muscles [21]. They are carried out according to the functional compromise of each limb and make it possible to maintain the corrections achieved with orthoses and reduce the need for surgery [21].

Kinesitherapy is not the only effective treatment technique for the improvement of these patients; it can be combined with other different therapies, such as hippotherapy [22] or aquatherapy [23]. This combination leads to improvements in the progression of gross motor skills [22] and motor function, speed of movement, trunk control, stability and, above all, functionality [23].

In addition to physiotherapy, surgical techniques and pharmacological interventions can be used to reduce pain, with beneficial results when carried out together [24].

Early treatment in these patients was shown to be necessary, as it helped to reduce the occurrence of possible complications and improved patient recovery. On the other hand, in most studies, patients with AMC were managed by a multidisciplinary team. Furthermore, only three studies [8,9,11] make direct mention of the importance of family involvement in the maintenance of rehabilitation treatment.

**Table 2:** Main characteristics of included studies.

Authors (Study Desing)	Participants	Intervention	Total Time	Variables	Results
Elfassy, C. et al. [20], 2020. (Qualitative study based on grounded theory)	n = 27 ± 14–21 years old. G1 ^1^: n = 6 ± Young people with arthrogryposis. G2 ^2^: n = 11 ± Carers. G3 ^3^: n = 10 ± Health professionals.	Interviews were conducted in person or by telephone and were digitally recorded and transcribed for later analysis.	12 months.	CMOP-E ^4^: Elements on physical, cognitive, affective, environmental, occupational, national performance and activity, activity domains and participation.	Rehabilitation is beneficial from early childhood to late adolescence, as it helps to determine future treatment. Early initiation of rehabilitation is necessary.
Gagnon, M. et al. [25], 2021. (Single cohort study)	n = 10 ± 8–21 years old.	Individualised exercise programme carried out at home, conducted remotely using telerehabilitation.	4 months.	APPT ^5^: Pain. GAS ^6^ PAQ-A ^7^: Physical activity. PODCI ^8^: Function. ROM ^9^: Joint range.	Statistically significant improvements were recorded for the pain and comfort domain, physical activity and function after intervention.
Valdés-Flores, M. et al. [18], 2016. (Cross-sectional study)	n = 50 ± 0–7 years old. n = 22 ± Men. n = 28 ± Women.	Specific rehabilitation and physiotherapy programmes for patients referred to the Genetics Department of the referred to the Genetics Department of the National Rehabilitation Institute of Mexico with a presumptive diagnosis of AMC.	36 months.	Variety of diagnostic tests: physical and radiographic examinations, pregnancy and delivery data, family medical history and karyotype.	The importance of such programmes and the need for a multidisciplinary approach to improve these patients were multidisciplinary approach to improve these patients.
Rojo Osuna, DJ. et al. [13], 2016. (Case series study)	n = 17 ± 10 months-16 years old n = 8 ± Men. n = 9 ± Women.	The records of patients with a diagnosis of AMC.	24 months.	Charting: To evaluate phenotypic characteristics reported in clinical records.	When arthrogryposis is diagnosed, treatment by a multidisciplinary team is essential. Amyloplasia is the most common type of AMC.
Gür, G. et al. [7], 2016. (Case report study)	n = 2. Case 1: 7-month-old baby. Case 2: 6-month-old infant.	Serial orthopaedic treatment was applied to reduce bilateral knee flexion contractures.	12 months.	GMFCS ^10^: Ambulatory capacity of children. Universal goniometry: Range of motion of joints.	Bilateral passive extension limitation improved; in the first case, the increase in passive extension range was 75°, and in the second case it was 45°.
Hernández Antúnez, N. et al. [14], 2015. (Case series study)	n = 19: n = 14 ± Men. n = 5 ± Women.	Physiotherapy and transfer training	60 months.	WeeFIM ^11^: Severity of disability and functionality in an objective manner. Data recording form: Sociodemographic and clinical variables, related to the treatments carried out and functionality.	Good scores were in cognitive and behavioural areas. Most of the children achieved independent walking, thanks to physiotherapy treatment.
Azbell, K. et al. [8], 2015. (Case report study)	n = 1 ± NB ^12^ 11-day-old.	Regular home (parents) and clinic (physiotherapist and occupational therapist) programme of stretching, strengthening, splinting, casting and bilateral Achilles tenotomies.	9 months.	PSFS ^13^: Functional changes and patient involvement. PDMS-2 ^14^: Fine and gross motor skills. Norkin method: passive ROM ^9^. FLACC ^15^: Pain.	Improvements were observed in all components of the ICF ^16^. Its total score improved by 2.34 points.
Ayadi, K. et al. [15], 2015. (Case series study)	n = 23 ± Average age of 6.6 years n = 13 ± Men. n = 10 ± Women.	The records of children with AMC in the orthopaedic department of the Habib-Bourguiba University Hospital Centre in Sfax (Tunisia) were reviewed. Treatments were not specified	144 months.	PODCI ^8^: Upper limb function, transfers and mobility, sport participation, pain, happiness and general function.	As a result of the treatments, an average functional score of 69.57 was obtained. Multidisciplinary care is necessary and should be provided early and continuously.
Águila Tejeda, G. et al. [9], 2013. (Case report study)	n = 1 ± 8-year-old girl.	Physiotherapy and psychotherapy (with family support). Rehabilitation was carried out at the CEPROMEDE ^17^. The physiotherapy treatment consisted of: breathing exercises, thermotherapy, massage, kinesitherapy, electrotherapy and adaptation to BADL ^18^.	72 months.	Morpho-functional assessment of the patient and evaluation of the results after the treatments applied.	Lower limb limitations improved by 80% with physiotherapy and rehabilitation treatment, as well as quality of life, ambulation and performance of BADL.
Binkiewicz-Glinska, A. et al. [10], 2013. (Case report study)	n = 1 ± NB 3-weeks-old.	Physiotherapy based on massage therapy, kinesitherapy (wrist and fingers), positional therapy, proprioception and sucking reflex stimulation	6 months.	ROM.	Improved range of motion and functionality of shoulder, elbow, wrist, hip and knee joints through early rehabilitation, comprehensive and multidisciplinary rehabilitation.
Beetar, P. [11], 2011. (Case report study)	n = 1 ± 2- month-old girl.	Physiotherapy with the help of the child’s mother. The routine consisted of kinesiotherapy, mat exercises for motor development, proprioception and gait training.	120 months.	Various diagnostic tests: X-rays, muscle biopsies, electrophysiological studies, genetic studies or magnetic resonance imaging.	Early initiation of physiotherapy preserved and restored joint mobility, muscle tone and proprioception.
Dillon, ER. et al. [19], 2009. (Cross-sectional study)	n = 26 ± 5–18 years old. G1 y G2: n = 8 ± Men. n = 5 ± Women. G1: n = 4 ± Distal arthrogryposis. n = 9 ± Amyoplasia. G2: n = 13 ± Typical development.	Young people with amyloplasia or distal or distal arthrogryposis, and youngsters with typical development of the same age and sex.	7 days.	Activity Monitor Step Watch 3: Frequency, duration, intensity of ambulatory activity and daily steps. Activity scale for children and performance questionnaires: Compares activity levels presented by the Step Watch 3.	Thanks to surgical interventions and rehabilitation, most of the children became ambulant, achieved relative independence in BADL and even attended school.
Taricco, LD. et al. [12], 2009. (Case report study)	n = 1 ± 35-year-old woman.	15 sessions of physiotherapy, 5 sessions of hydrotherapy, 2 sessions of occupational therapy, 2 sessions of psychotherapy and 1 session of art therapy. 45 minutes 5 days a week.	15 months.	VAS ^19^: Pain. Universal Goniometer: Range of motion of joints.	It is essential that orthopaedic and rehabilitative treatment and planning be carried out by an interdisciplinary team.
Morcuende, JA. et al. [16], 2008. (Case series study)	n = 16 ± 10 months-12 years old n = 11 ± Men. n = 5 ± Women.	Records of patients with clubfoot associated with arthrogryposis are reviewed. Ponseti’s method was performed in all these patients.	144 months.	Patient’s age at first visit, previous treatment, number of casts used, possible surgeries and degree of ankle dorsiflexion after tenotomy were assessed.	The Ponseti method is very effective for early correction of clubfoot associated with arthrogryposis; it reduces the need for extensive corrective surgeries or talectomies.
De Miguel Benadiba, C. et al. [17], 1992. (Case series study)	n = 24 ± Average age 11.1 years n = 14 ± Men. n = 10 ± Women.	Physiotherapy by means of kinesitherapy and stretching, which were used before and after orthopaedic treatment.	156 months.	Patient or family survey: functional capacity and social integration of patients.	Most patients become independent and able to advocate for themselves when they reach adulthood, thanks to early initiation of multidisciplinary treatment and family support.

G1 ^1^: Group 1; G2 ^2^: Group 2; G3 ^3^: Group 3; CMOP-E ^4^: Canadian of Occupational Performance and Engagement; APPT ^5^: Pain in adolescents and children; GAS ^6^: Goal achievement; PAQ-A ^7^: Physical activity levels in the last 7 days; PODCI ^8^: Paediatric Outcomes Data Collection Instrument; ROM ^9^: Range of Movement; GMFCS ^10^: Gross Motor Function Classification System; WeeFIM ^11^: Functional Independence Measure; NB ^12^: New Born; PSFS ^13^: Patient Specific Functional Scale; PDMS-2 ^14^: Peabody Developmental Motor Scales; FLACC ^15^: Face, Legs, Activity, Cry, and Consolability; ICF ^16^: International Classification of Functioning, Disability and Health; CEPROMEDE ^17^: Provincial Sports Medicine Centre; BADL ^18^: Basic Activities of Daily Living; VAS ^19^: visual analogue scale.

### 3.3. Methodological Quality Synthesis

Methodological quality assessed using the critical appraisal JBI tools. The specific results for each of the study designs can be found in Table 3, Table 4, Table 5, Table 6 and Table 7. 

As shown in Table 3, most of the case reports showed a good description of the case, but some of them only mention the type of intervention and do not specify the intensity, duration or frequency, which makes it difficult to reproduce the results. On the other hand, none of the case studies reported adverse effects.

The case series design studies showed a low methodological quality, especially the studies by De Miguel Benadabia et al. [17] and Rojo Osuna et al. [13], in which most of the items were not explicitly specified. In addition, as shown in Table 4, item 9 was the only item in which none of the studies specified the geographical characteristics of the participants directly, making it difficult to extrapolate the results in future studies.

With regard to the cross-sectional design studies, it should be noted that none of them adequately identify the confounding factors or how to address them (Table 5).

Only the study by Elfassy et al. [20] presented a qualitative design whose main methodological conflict was not stating how participants’ beliefs or values could influence the results (Table 6). Moreover, the Table 7 shows the result of the quality appraisal for cohort study of Gagnon et al [21]. 

## 4. Discussion

This systematic review provides an overview of the state of the art of the different therapeutic approaches in rehabilitation and the benefits that physiotherapy can provide to patients with AMC.

Treatment interventions are very varied in the selected studies. Physiotherapy and/or rehabilitation programmes stand out [8,9,10,11,12,14,16,17,18,25]. These, in some cases, are delivered remotely via telerehabilitation [25]. These interventions are not always applied by the physiotherapist or rehabilitation doctor [9,12,14,16,17,18,19], but by the family environment [11,25]. This is due to the use of telerehabilitation. Sometimes, the intervention is accompanied by orthopaedic treatment, either conventional or surgical, to reduce or avoid the increase of contractures [7,15]. In other cases, it is accompanied by psychotherapy, occupational therapy and art therapy [12]. Rehabilitation and physiotherapy interventions consist mainly of kinesitherapy, which is mobilisations through specific therapeutic exercise programmes [25], stretching, strengthening [8], breathing exercises [9], hydrotherapy [12], thermotherapy [8], massage [8], electrotherapy, exercises to improve motor development [11] and adaptation to activities of daily living [9]. It was possible to verify that all patients who were treated by rehabilitation achieved an improvement in terms of joint range, were able to walk independently, perform activities of daily living on their own, reduce the degree of possible limitations and achieve improvements in all the components proposed by the ICF [7,8,9,14,19]. This is in addition to orthopaedic treatment, the Ponseti method [16,26] and, in some cases, surgery. It must be kept in mind that conservative treatment is limited in certain severe contractures very present in AMC [26]. 

Rehabilitation and physiotherapy interventions consist mainly of kinesitherapy, which is mobilisations through specific therapeutic exercise programmes, stretching, strengthening, breathing exercises, hydrotherapy, thermotherapy, massage, electrotherapy, exercises to improve motor development and adaptation to activities of daily living.

Furthermore, after evaluating the studies, all agree that rehabilitation provides beneficial results in patients with AMC. It is important to note that not all of them provided the same treatment protocol, but that certain modifications were observed in each of the studies. Despite this, each of the techniques employed achieved good results. However, it should be noted that there are gaps in the literature on AMC that indicate the need for further studies to establish more information on the evidence-based treatment of patients with arthrogryposis [4].

Several studies have concluded that physiotherapeutic treatment is indispensable in these patients [4,14,15,18,19,25]. In fact, future lines of research are oriented towards the use of telerehabilitation to provide therapeutic intervention at home [25], although this proposal needs to be studied in depth. Kinesitherapy in all its forms (passive, active or self-assisted), despite being one of the most widely used techniques in the therapeutic approach to people with AMC, does not have a specific protocol for these patients [4,21]. Future clinical trials need to define kinesitherapy interventions in detail in order to achieve solid evidence for this useful therapeutic strategy, which can be applied at all ages without adverse effects.

Furthermore, treatments must be carried out continuously over time in order to achieve the maximum possible autonomy and facilitate the social integration of patients [15]. In most cases, patients expect visible results in a short period of time, become discouraged and stop attending their treatment sessions, thus reducing the effectiveness of the treatment [15]. For this reason, some patients drop out of treatment before completing all the sessions necessary to achieve adequate recovery, to the point of relapse [27]. 

Rehabilitation should be carried out early [10,11,12,15,20,25,27]. This will help patients to regain mainly joint mobility, muscle power and proprioception (14). This can, in turn, be beneficial in determining future treatments more easily [20]. It also allows for a more successful recovery, always within limits, an improvement in quality of life [9,17] and a decrease in the risk of future complications and deformities [27], among others. On the other hand, correction of deformities can satisfactorily achieve ambulation, even in adults [12].

Treatment should be carried out by a multidisciplinary team, as they require treatment from different aspects, not only from the point of view of physiotherapy [10,12,13,15,17,18]. The communication and coordination of each of the specialists in charge of each case makes the treatment of patients much more complete, beneficial and, to a certain extent, reduces recovery time [18]. On the other hand, it should be noted that in most of our studies, the family is the cornerstone of treatment, together with physiotherapists, occupational therapists, psychologists and nurses.

It is essential that treatment is specific and individualised for each patient [11,18]. We must define personalised therapeutic objectives, as this will allow better benefits to be achieved in terms of the evolution of the treatment [28]. This aspect is valid and useful for all disciplines, not only for physiotherapy. It must be taken into account that each treatment must be carried out according to the needs of the patients [18], as no two patients have exactly the same symptomatology [8]. For this reason, we cannot use the same treatment protocol for all patients diagnosed with arthrogryposis [27].

Future lines of research, apart from telerehabilitation and those described above, should be considered. Surgical options, such as posterior spinal fusion in concomitant scoliosis, are proposed for the prevention of lung function impairment [29,30], and other surgeries in cases of severe contractures [26], considering the specific difficulties of this treatment and of surgery in general in ACM [31], and specific therapies with nerve and muscle stem cells are proposed [32]. 

The scientific literature describes family support and involvement as a key element in the evolution and improvement of children with chronic problems [33]. In fact, the family should be considered as a branch of the multidisciplinary team caring for children with AMC; in most cases, they will be the ones who will be able to provide daily care. With this information in mind, direct communication between healthcare professionals and the family and education of the family members will be necessary to empower them [34].

The role of the family or the closest environment is also fundamental [8,15,20]. It is important that, in addition to the patient him/herself, the people who live with the patient support and reinforce the treatment [7,9,35,36]. This will benefit the individual and his or her environment, favouring the application of a holistic treatment, which is fundamental in CMA [20]. This additional support to rehabilitative treatment has been studied not only in arthrogryposis, but also in other conditions. In all of them, benefits of various kinds have been seen, such as in motor development [37,38,39,40] and control [35,38], psycho-social [36] and cost reduction [41]. In other cases, these benefits have not been conclusively demonstrated, such as in Developmental Coordination Disorder [42]. Interventions that can produce these benefits include massage [43,44], specific [37,40,45] and global mobilisations [35,38,39] learnt from health professionals, orthopaedic tools management [45] and psychological support [35,36]. All of this will improve independence [9,11,46] and favour relations with their social environment [9,15,20,47]. 

### Limitations and Strengths

This systematic review has limitations. Firstly, the methodological quality of the included studies directly influences the results of the review. The results of the review should be viewed with caution as the rehabilitation programmes and the characteristics of the participants should have been further defined in order for the results to be extrapolated to the general population. There is a need for standardisation in reporting these data in future studies. Due to the limited number of investigations, all studies where reference was made to rehabilitation or physiotherapy were included. Secondly, meta-analysis was not possible due to the heterogeneity of study designs and variables. Future research should be based on high quality methodologies. Increasing the number and quality of studies will allow for reliable results. Furthermore, the study sample is too small to obtain solid results, so it is necessary to increase the scientific production in order to know which is the best therapeutic strategy for these patients. However, in this pathology, it may be justified due to its low prevalence. Case study designs in rare diseases may be best suited to provide novel information as well as specific side effects of interventions. However, in this pathology, it may be justified due to its low prevalence [48]. Case study designs in rare diseases may be best suited to provide novel information as well as specific side effects of interventions. As stated by Sampayo Cordero, case studies are important for systematic reviews of rare diseases to synthesise the state of the literature and provide clinically valuable information [49]. In our case, no clinical trials were included due to the absence of such trials in the scientific literature. In addition, it was not possible to use the GRADE tool to determine the degree of evidence because we did not present a meta-analysis.

On the other hand, among the strengths of the study is the use of the same methodological quality assessment tool to avoid the use of different types of scales. Another strength of the study is that no language restrictions were placed so that potential studies were not excluded.

## 5. Conclusions

The use of rehabilitation techniques for the treatment of people diagnosed with AMC provides satisfactory results.

Treatment must be specific, personalised and congruent with the needs of each patient. It must also be constant and long-lasting, since, in most cases, it must be maintained for the rest of their lives.

It is recommended that it is applied as early as possible, as it can help to reduce the risk of complications of arthrogryposis.

At the same time, it is essential that these patients are treated by a professional multidisciplinary team, where physiotherapy is essential in combination with other treatments to achieve results that improve the patient’s quality of life and state of health.

There is a need to increase the number of studies in this pathology. More clinical trials should be conducted to provide sufficient theoretical and clinical information. Studies should be of high quality and with a larger number of participants. At the same time, the study variables, measurement instruments, interventions applied to participants and methods of data analysis should be homogenised.

## Figures and Tables

**Table 1:** Complete search strategy and results.

Database	Results	Filters	Reviewed Articles	Selected Articles
PubMed ^1^	(“Arthrogryposis” [Mesh]) AND “Physical Therapy Modalities” [Mesh])			
	64	Type of study	2	1
	((“Arthrogryposis” [Mesh]) AND “Physical Therapy Modalities” [Mesh]) AND “Contracture” [Mesh])			
	12	-	12	0
	((“Arthrogryposis” [Mesh]) AND “Rehabilitation” [Mesh])			
	103	Type of study	12	1
	((“Arthrogryposis” [Mesh]) AND “Rehabilitation” [Mesh]) AND “Joints” [Mesh]			
	34	Type of study	34	0
	((“Arthrogryposis” [Mesh]) AND “Physical Therapy Modalities” [Mesh]) AND “Clubfoot” [Mesh]			
	10	Type of study	4	0
	“Arthrogryposis AND Physical Therapy”			
	136	Type of study	4	2
	“Physiotherapy in arthrogryposis”			
	971	Type of study	15	1
	Arthrogryposis multiplex congenita AND treatment			
	376	Type of study	149	4
	(Arthrogryposis OR “Arthrogryposis multiplex congénita” OR Artrogriposis OR “Artrogriposis múltiple congénita”) AND (“Physical Therapy Modalities” OR Rehabilitation OR Physiotherapy OR Fisioterapia OR treatment OR “physical therapy” OR Rehabilitación OR Tratamiento OR Rehab* OR Fisio*) AND (Contracture OR Joints OR Clubfoot OR contractura)			
	183	Type of study	3	0
ScienceDirect ^2^	“Arthrogryposis AND Physical Therapy”			
	1.385	Type of study Subject areas	302 20	2
	(Arthrogryposis OR “Arthrogryposis multiplex congénita” OR Artrogriposis OR “Artrogriposis múltiple congénita”) AND (“Physical Therapy Modalities” OR Rehabilitation OR Physiotherapy OR Fisioterapia OR treatment OR “physical therapy” OR Rehabilitación OR Tratamiento OR Rehab* OR Fisio*) AND (Contracture OR Joints OR Clubfoot OR contractura)			
	0		0	0
Scielo ^3^	Fisioterapia en artrogriposis			
	2	-	0	1
	Arthrogryposis in physical therapy			
	1	-	0	1
	(Arthrogryposis OR “Arthrogryposis multiplex congénita” OR Artrogriposis OR “Artrogriposis múltiple congénita”) AND (“Physical Therapy Modalities” OR Rehabilitation OR Physiotherapy OR Fisioterapia OR treatment OR “physical therapy” OR Rehabilitación OR Tratamiento OR Rehab* OR Fisio*) AND (Contracture OR Joints OR Clubfoot OR contractura)			
	0	-	0	0
Scopus ^4^	“Artrogriposis”			
	69	Type of study	15	0
	“Artrogriposis y fisioterapia”			
	2	-	0	1
	(Arthrogryposis OR “Arthrogryposis multiplex congénita” OR Artrogriposis OR “Artrogriposis múltiple congénita”) AND (“Physical Therapy Modalities” OR Rehabilitation OR Physiotherapy OR Fisioterapia OR treatment OR “physical therapy” OR Rehabilitación OR Tratamiento OR Rehab* OR Fisio*) AND (Contracture OR Joints OR Clubfoot OR contractura)			
	568	Subject area	15	0
WOS ^5^	Arthrogryposis AND physical therapy			
	111	Type of study	30	0
	(Arthrogryposis OR “Arthrogryposis multiplex congénita” OR Artrogriposis OR “Artrogriposis múltiple congénita”) AND (“Physical Therapy Modalities” OR Rehabilitation OR Physiotherapy OR Fisioterapia OR treatment OR “physical therapy” OR Rehabilitación OR Tratamiento OR Rehab* OR Fisio*) AND (Contracture OR Joints OR Clubfoot OR contractura)			
	510	Research areas	38	0
ENFISPO ^6^	(Artrogriposis OR Artrogriposis–Fisioterapia OR Artrogriposis–Rehabilitacion OR Artrogriposis–Tratamiento OR Artrogriposis en niños–Fisio OR Artrogriposis en niños–Rehab)			
	5	-	0	1
	(Arthrogryposis OR “Arthrogryposis multiplex congénita” OR Artrogriposis OR “Artrogriposis múltiple congénita”) AND (“Physical Therapy Modalities” OR Rehabilitation OR Physiotherapy OR Fisioterapia OR treatment OR “physical therapy” OR Rehabilitación OR Tratamiento OR Rehab* OR Fisio*) AND (Contracture OR Joints OR Clubfoot OR contractura)			
	0		0	0
JSTOR ^7^	Arthrogryposis AND Physical Therapy			
	41	Type of study	32	0
	(Arthrogryposis OR “Arthrogryposis multiplex congénita” OR Artrogriposis OR “Artrogriposis múltiple congénita”) AND (“Physical Therapy Modalities” OR Rehabilitation OR Physiotherapy OR Fisioterapia OR treatment OR “physical therapy” OR Rehabilitación OR Tratamiento OR Rehab* OR Fisio*) AND (Contracture OR Joints OR Clubfoot OR contractura)			
	0		0	0
Google Schoolar	Fisioterapia en artrogriposis			
	401	Type of study	386	0
	(Arthrogryposis OR “Arthrogryposis multiplex congénita” OR Artrogriposis OR “Artrogriposis múltiple congénita”) AND (“Physical Therapy Modalities” OR Rehabilitation OR Physiotherapy OR Fisioterapia OR treatment OR “physical therapy” OR Rehabilitación OR Tratamiento OR Rehab* OR Fisio*) AND (Contracture OR Joints OR Clubfoot OR contractura)			
	523	Publication year	313	0
UCA Library ^8^	Artrogriposis múltiple congénita tratamiento			
	39	Type of study	31	0
	Artrogriposis y contractura			
	47	Type of study	39	0
	(Arthrogryposis OR “Arthrogryposis multiplex congénita” OR Artrogriposis OR “Artrogriposis múltiple congénita”) AND (“Physical Therapy Modalities” OR Rehabilitation OR Physiotherapy OR Fisioterapia OR treatment OR “physical therapy” OR Rehabilitación OR Tratamiento OR Rehab* OR Fisio*) AND (Contracture OR Joints OR Clubfoot OR contractura)			
	0		0	0
ProQuest Research Library	Artrogriposis			
	249	Type of study	26	0
	Physical therapy in arthrogryposis			
	3.070	Type of study	444	0
	(Arthrogryposis OR “Arthrogryposis multiplex congénita” OR Artrogriposis OR “Artrogriposis múltiple congénita”) AND (“Physical Therapy Modalities” OR Rehabilitation OR Physiotherapy OR Fisioterapia OR treatment OR “physical therapy” OR Rehabilitación OR Tratamiento OR Rehab* OR Fisio*) AND (Contracture OR Joints OR Clubfoot OR contractura)			
	1.159	Tipo de estudio	58	0
Cochrane Library	Arthrogryposis			
	0	-	0	0
	(Arthrogryposis OR “Arthrogryposis multiplex congénita” OR Artrogriposis OR “Artrogriposis múltiple congénita”) AND (“Physical Therapy Modalities” OR Rehabilitation OR Physiotherapy OR Fisioterapia OR treatment OR “physical therapy” OR Rehabilitación OR Tratamiento OR Rehab* OR Fisio*) AND (Contracture OR Joints OR Clubfoot OR contractura)			
	3	-	0	0
EBSCO ^9^	Artrogriposis			
	0	-	0	0
	(Arthrogryposis OR “Arthrogryposis multiplex congénita” OR Artrogriposis OR “Artrogriposis múltiple congénita”) AND (“Physical Therapy Modalities” OR Rehabilitation OR Physiotherapy OR Fisioterapia OR treatment OR “physical therapy” OR Rehabilitación OR Tratamiento OR Rehab* OR Fisio*) AND (Contracture OR Joints OR Clubfoot OR contractura)			
	0	-	0	0
PEDro ^10^	Arthrogryposis			
	0	-	0	0
	(Arthrogryposis OR “Arthrogryposis multiplex congénita” OR Artrogriposis OR “Artrogriposis múltiple congénita”) AND (“Physical Therapy Modalities” OR Rehabilitation OR Physiotherapy OR Fisioterapia OR treatment OR “physical therapy” OR Rehabilitación OR Tratamiento OR Rehab* OR Fisio*) AND (Contracture OR Joints OR Clubfoot OR contractura)			
	0		0	
TOTAL	10.074	-	1.984	15

Pubmed ^1^: National Library of Medicine; ScienceDirect ^2^: Elsevier’s database of bibliographic references and citations; Scielo ^3^: Scientific Electronic Library Online; Scopus ^4^: Elsevier’s database of bibliographic references and citations; WOS ^5^: Web of Science; ENFISPO ^6^: Database of bibliographic references and citations of the Library of the Faculty of Nursing, Physical Therapy and Podiatry of the Complutense University of Madrid; JSTOR ^7^: Journal Storage; UCA Library ^8^: Database of bibliographic references and citations of the Library of the Faculty of Nursing, Physical Therapy of the University of Cadiz; EBSCO ^9^: Elton Bryson Stephens Company; PEDro ^10^: Physiotherapy Evidence Database 7.

**Table 3:** Methodological quality assessment of case report studies through the JBI quality appraisal tool.

JBI Items/Studies	Aguila Tejada et al., 2013 [9]	Azbell et al., 2015 [8]	Beetar et al., 2011 [11]	Binkiewicz-Glinska et al., 2013 [10]	Gür et al., 2016 [7]	Taricco et al., 2009 [12]
1. Patients’ characteristics	Y	Y	Y	Y	Y	Y
2. History and timeline	Y	Y	Y	Y	Y	Y
3. Current clinical condition	Y	Y	Y	Y	Y	Y
4. Assessment methods	Y	Y	Y	Y	Y	Y
5. Treatment description	U	Y	U	Y	U	Y
6. Post-intervention clinical condition	Y	Y	Y	Y	Y	Y
7. Adverse events described	N	N	N	N	N	N
8. Takeaway lessons	N	Y	Y	Y	Y	Y

Note: Y = yes; N = no; U = nuclear; NA = not applicable.

**Table 4:** Methodological quality assessment of case series studies through the JBI quality appraisal tool.

JBI Items/Studies	Ayadi et al., 2015 [15]	De Miguel Benadabia et al., 1992 [17]	Hernández Antúnez et al., 2015 [14]	Morcuende et al., 2008 [16]	Rojo-Osuna et al., 2016 [13]
1. Inclusion criteria	Y	U	Y	U	U
2. Reliable condition measure	Y	U	Y	Y	U
3. Methods for identification of condition	Y	U	Y	U	U
4. Consecutive inclusion	Y	U	Y	Y	U
5. Complete inclusion	Y	U	Y	U	U
6. Clear demographics of participants	Y	Y	Y	Y	U
7. Clear reports of outcomes	Y	Y	Y	Y	Y
8. Follow-up results	U	U	U	Y	U
9. Report of clinic demographic information	U	U	U	U	U
10. Appropriate statistical analysis	Y	U	U	U	U

Note: Y = yes; N = no; U = nuclear; NA = not applicable.

**Table 5:** Methodological quality assessment of cross-sectional studies through the JBI quality appraisal tool.

JBI Items/Studies	Dillon et al., 2009 [19]	Valdes-Flores et al., 2016 [18]
1. Inclusion criteria	Y	U
2. Reliable condition measure	Y	Y
3. Methods for identification of condition	Y	Y
4. Consecutive inclusion	Y	Y
5. Complete inclusion	U	U
6. Clear demographics of participants	U	U
7. Clear reports of outcomes	Y	Y
8. Follow-up results	Y	Y

Note: Y = yes; N = no; U = nuclear; NA = not applicable.

**Table 6:** Methodological quality assessment of qualitative study through the JBI quality appraisal tool.

JBI Items/Studies	Efassy et al., 2009 [20]
1. Congruency between stated philosophy and methodology	Y
2. Congruency between methodology and research question	Y
3. Congruency between methodology and data collection	Y
4. Congruency between methodology and representation and data analysis	Y
5. Congruency between methodology and results	N
6. Locating research culturally or theoretically	N
7. Participants’ voice represented	Y
8. Ethical approval	Y
9. Conclusion drawn from analysis, interpretation or data	Y

Note: Y = yes; N = no; U = nuclear; NA = not applicable.

**Table 7:** Methodological quality assessment of single cohort study through the JBI quality appraisal tool.

JBI Items/Studies	Gagnon et al., 2021 [25]
1. Similar groups at baseline and same population recruitment	Y
2. Exposure measured similarly	Y
3. Valid and reliable measurement of exposure	U
4. Confounding factors identification	Y
5. Strategies to deal with confounding factors stated	Y
6. Participant’s free of the outcome at the start of the study	N
7. Valid and reliable measurement of exposure	U
8. Enough time for the outcome to occur	Y
9. Follow-up results	U
10. Strategies to address incomplete follow-up	U
11. Appropriate statistical analysis	Y

Note: Y = yes; N = no; U = nuclear; NA = not applicable.

## Data Availability

Not applicable.
